# Is there a difference in the spread of excitation at different electrode locations along the cochlea?

**DOI:** 10.1590/2317-1782/e20240090en

**Published:** 2025-10-20

**Authors:** Georgea Espindola Ribeiro, Ana Tereza de Matos Magalhães, Maria Valéria Schmidt Goffi Gomez, Carolina Gianzantti da Costa, Robinson Koji Tsuji, Rubens Vuono de Brito

**Affiliations:** 1 Departamento de Oftalmologia, Otorrinolaringologia e Cirurgia de Cabeça e Pescoço, Faculdade de Medicina, Universidade de São Paulo – USP - São Paulo (SP), Brasil.

**Keywords:** Hearing, Deafness, Cochlear Implant, Telemetry, Electrodes, Neural Response, Spread of Excitation, Channel Interaction

## Abstract

**Purpose:**

To identify whether there are differences in the amplitude and width of spread of excitation (SOE) across the apical, medial and basal regions of the cochlea.

**Methods:**

Cross-sectional retrospective study approved by the Ethics Committee of the institution. The study included adults with postlingual deafness, undergoing cochlear implant (CI) surgery, with present intraoperative neural responses in which the SOE was investigated in the basal (6), medial (11) and apical (16) electrodes. Neural response telemetry thresholds, peak amplitudes (µv) of the SOE function and SOE width in millimeters were collected and grouped by the electrode array type for analysis using the Mann Whitney and Kruskal Wallis tests.

**Results:**

Seventy-one subjects were selected, 27 with perimodiolar array and 44 with straight array. There were no significant differences in the peak amplitudes among evaluated electrodes in both groups. However, SOE width (mm) of the medial electrode was significantly wider in both arrays, followed by the width of the basal electrode.

**Conclusion:**

Although the SOE amplitude was similar suggesting similar neural recruitment in different regions of the cochlea, wider spread was found in the medial region even in the perimodiolar array. Thus, the use of objective tests will become increasingly important to assist in CI mapping, aiming for more effective and individualized programming.

## INTRODUCTION

Cochlear implant (CI) candidacy is established for individuals with bilateral severe to profound sensorineural hearing loss who derive no benefit from conventional hearing aids^(1)^. The CI comprises an electrode array inserted into the scala tympani, situated beneath the basilar membrane and osseous spiral lamina^(2)^.

Electrodes within the cochlea deliver electrical current to the viable fibers of the auditory nerve, transmitting tonotopic information across the cochlea about the spectral attributes of the acoustic signal^(3)^. Loudness perception is mediated by the quantity of activated fibers (spatial summation) and the firing rate of neural impulses (temporal summation)^((4)^. Consequently, accurate electrode placement and proximity to the modiolus are critical for effective electrical stimulation^(5-7)^.

Pfingst et al.^(8)^ have demonstrated that both the cochlear anatomy and the distribution of remaining spiral ganglion cells vary along its length (basal, medial and apical). Thus stimulation thresholds may vary along the array due to differential neural responsiveness at each position as well as the electrode distance to the modiolus.

Recording the compound action potential of the auditory nerve (eCAP) via neural response telemetry (NRT) enables investigation of neural fiber behavior at different electrode positions, through measuring the eCAP threshold and advanced metrics like Spread of Excitation (SOE)^(6,9,10)^.

**Figure 1:**
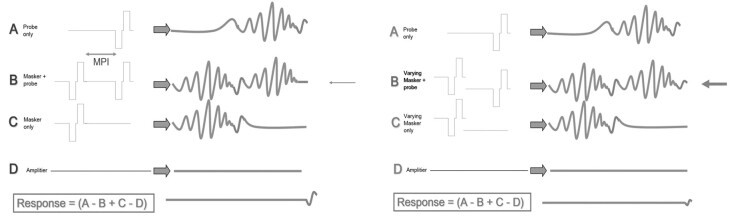
Diagram of SOE with prior masking technique adapted from Abbas et al.^(12)^

The SOE is assessed using a forward-masking paradigm, in which the masker stimulus is varied across electrodes while the probe and recording electrodes remain fixed. Plotting the recorded response amplitude as the masker shifts produces the SOE function^(3,11)^. Using subtraction paradigms, the subtracted response is maximal when masker and probe recruit the same fibers. The amplitude diminishes as the masker electrode moves away, since the stimulated fibers are not in refractory and are thus unstimulated^(12)^. The curve peaks around the probe electrode, where masker and probe coincide (Figure 1)^(12).^

Caption: (1) Right panel, in trace A, the test stimulus is presented and generates both the artifact and the neural response. In B, the masker is presented with a sufficient interval (MPI - masker-probe interval) so that when the test stimulus is presented, the fibers are in a refractory period. The result is masker and probe artifacts and a masker-only response. In C, the masker stimulus is presented and generates both an artifact and a neural response. In D, no stimulation is applied, only the amplifier is turned on. Subtraction of the tracings results only in the neural response to the test stimulus; (2) Left panel, when the masker and test electrodes are separated from each other, the fibers responding to the test are expected to be different from those responding to the masker, so they will not be in a refractory period after masking. The final amplitude after subtraction will become smaller as the masker becomes more distant

Ideally, each intracochlear electrode stimulates a focused number of neural fibers; however, the resultant electric field may spread within the cochlea, activating distant fibers^(3,11)^. Excessive current spread can compromise spectral precision delivered to the auditory nerve^(13)^.

Clinically, wide current spread can impair CI users’ recognition of vowels and consonants, affect pitch perception, loudness sensation, and current levels needs all of which may limit auditory rehabilitation outcomes^(14)^. Therefore, characterizing current spread at different points along the electrode array may reveal cochlear regions with reduced or absent excitable neurons, facilitating individualized optimization of programming parameters.

Another factor influencing SOE is electrode array design. While Kopsch et al.^(15)^ found no difference in SOE between straight and perimodiolar arrays, their study included only two straight-array subjects versus 57 with perimodiolar arrays. In contrast, Kim et al.^(16)^ reported broader SOE with straight arrays compared to perimodiolar ones, suggesting reduced neural overlap and channel interaction in perimodiolar designs. Berg et al.^(17)^, measuring SOE intraoperatively in users of both designs, observed widening SOE with increased electrode modiolus distance but only for perimodiolar arrays. Conversely, straight arrays showed increased current spread with deeper insertion.

Regarding SOE values at various cochlear regions, Xi et al.^(13)^ and Rader et al.^(18)^ observed greater current spread in the apical region than the basal region, attributed to its more tapered spiral geometry and higher neuronal density.

By contrast, Söderqvist et al.^(14)^ have observed wider SOE width in the medial region when compared to the basal and apical regions for straight arrays. The basal region presented the narrowest width of the three studied regions. The authors attributed these findings to the relative size of the scala tympani and possible poor conductance of the surrounding tissues, leading to current loss through the round window and rapid electric field decay.

These findings suggest that different electrode designs may yield distinct SOE profiles. Therefore, the objective of the present study was to identify whether there are differences in the SOE width and amplitude across the apical, medial, and basal regions of the cochlea, for both straight and perimodiolar electrode arrays.

## METHODS

This retrospective cross-sectional study was approved by the Institutional Ethics Committee (CAAE 03409212.8.0000.0068). All participants provided informed consent.

Data were obtained from medical records and intraoperative recordings of individuals implanted by the Cochlear Implant Group at Hospital das Clínicas, University of São Paulo.

### Study sample

Intraoperative records from January 2016 to June 2021 were selected based on inclusion criteria: adults (≥ 18 years) with postlingual hearing loss who received Cochlear™ Nucleus® devices with perimodiolar arrays (CI 24RE CA, CI 532, CI 632) or straight arrays (CI 422, CI 522, CI 622); and who had recorded NRT thresholds and SOE at electrodes 6, 11, and 16 using a forward-masking protocol^(12,19)^.

Exclusion criteria included neuropathy, cochlear malformations, facial nerve stimulation during NRT, absent NRT, partial array insertion, or electrode tip fold over.

Data collected included participant age at surgery, etiology, onset of deafness, and array type. Using Custom Sound® EP software, tNRT threshold, eCAP peak amplitude (µV), SOE width measured at 0.75 of the curve (mm), and current levels were extracted.

### Procedures

CustomSound™ EP 3.0 software connected to an interface (Pod) and a Nucleus® 5 or newer speech processor (Cochlear™) allowed the eCAP recordings. Immediately post-insertion under anesthesia, CI integrity was assessed via impedance telemetry, followed by NRT (tNRT and SOE function).

Pulse trains were delivered to intracochlear electrodes 6 (basal), 11 (medial), and 16 (apical), and the response recorded at an adjacent electrode. The eCAP waveform comprised a negative N1 peak followed by a positive P1 peak. Amplitude was defined as the voltage difference between N1 and P1. The software applied linear regression to the amplitude growth function to determine the neural response threshold (tNRT) and its slope for each electrode; the threshold was identified as the lowest current producing a neural response^(12)^.

**Figure 2:**
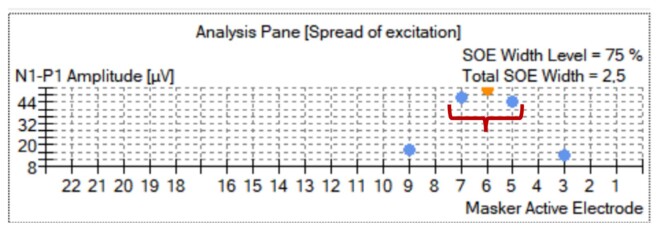
Custom Sound EP software print screen showing the SOE recording, with the measurement of the dispersion width calculated in millimeters (mm) at the 75% point of the curve. The horizontal key has been drawn to illustrate the measured width, in this example, 2.5 mm

The SOE protocol used at least 10 current levels (CL) above tNRT^(20)^, with a stimulation rate of 40 Hz and a masker-probe interval of 400 µs. Data was composed of the peak amplitude of the function (µv), spread of excitation width in millimeters (mm) at the 75% transection point of the curve, and the current level used to record the SOE (Figure 2).

The sample selection was conducted by convenience sampling, including all implanted participants who met the inclusion criteria during the study period.

For statistical analysis, straight and perimodiolar groups were compared using the nonparametric Mann–Whitney test for independent samples. For comparison across different regions along the electrode array (6, 11, and 16), the Kruskal-Wallis test was used, with Dunn's test for post-hoc multiple comparisons using BioEstat 5.0 software, adopting a significance level of 5% in all tests^((21,22))^.

## RESULTS

Of 177 intraoperative records, 71 met inclusion criteria. Demographic distribution is presented in Table1.

**Table 1:** Demographic distribution of the sample studied

	Straight Array (n=44)	Perimodiolar Array (n=27)
Average age (years)	52 (min 19/max 81)	42 (min 19/max 74)
Electrode Array (N)		
Straight Array		
CI 422	40	-
CI 522	3	-
CI 622	1	-
Perimodiolar Array		
CI 532	-	14
CI 24RECA	-	12
CI 632	-	1
Etiology (N)		
Unknown	27	14
Otosclerosis	5	4
Meningitis	4	-
Traumatic Head Injury	3	2
Ototoxicity	2	1
Infection		2
Mondini	-	2
Rubella	1	1
Autoimmune	1	-
Sudden hearing loss	-	1
Chronic otitis media	1	-

Caption: N = number of ears; min = minimum; max = maximum

Significant difference was found in the tNRT thresholds between straight and perimodiolar arrays only at electrode 6 (basal). Amplitude did not differ across array types in any cochlear region. SOE width was significantly different between electrode arrays, showing wider dispersion of excitation in the straight arrays for all regions of the cochlea (Table2).

**Table 2:** Median of the variables studied and comparison between the straight and perimodiolar bundles using the Mann-Whitney test

	Straight Array (N = 44)	Perimodiolar Array (N = 27)	p*
tNRT (CL)	Med (min/max)	Med (min/max)	
E16	191 (142 – 232)	184 (162 – 209)	0.3816
E11	200 (177 – 234)	202 (165 – 223)	0.6608
E6	206 (183 – 237)	198 (155 – 218)	0.0050*
SOE amplitude (μV)			
E16	83.33 (11.95 – 265.71)	66.94 (12.98 – 233.95)	0.5242
E11	68.31 (10.24 – 234.63)	67.62 (10.59 – 200.14)	0.6109
E6	67.62 (18.10 – 283.81)	57.03 (20.49 – 209.36)	0.3769
SOE width (mm)			
E16	2.31 (0.88 – 5.31)	1.58 (0.59 – 4.38)	**0.0007** *
E11	3.30 (1.01 – 8.06)	2.44 (0.59 – 3.73)	0.0003*
E6	2.02 (0.63 – 6.69)	1.68 (0.69 – 3.12)	0.0429*

* *p* value test of Mann Whitney

Caption: N = number of ears; Med = median; min = minimum; max = maximum; E16 = apical electrode 16; E11 = medial electrode 11; E6 = basal electrode 6; tNRT = threshold Neural Response Telemetry; SOE = *Spread of excitation*; CL = current level; mm = millimeters; μV = microvolts

Within each array type, tNRT thresholds were significantly lower at apical electrode 16 compared to 6 and 11 (Table3). Peak SOE amplitudes did not differ significantly across electrodes for either array (Table3).

**Table 3:** Results of the parameters studied in the groups implanted with straight and perimodiolar arrays, analyzed using the Kruskal Wallis test

	E16	E11	E6	p*
tNRT (CL)	Med (min/max)	Med (min/max)	Med (min/max)	
Straight Array	189 (142 – 232)*	200 (156 – 235)	205 (183 – 237)	**<0.0001**
Perimodiolar Array	186 (155 – 209)	194 (159 – 229)	197 (155 – 221)	0.1281
SOE peak amplitude (μV)				
Straight Array	95 (21 – 265)	69 (19 – 271)	67 (24 – 300)	0.6181
Perimodiolar Array	65 (12 – 256)	68 (14 – 247)	56 (21 – 209)	0.6481
SOE width (mm)				
Straight Array	2.34 (0.88 – 5.31)	2.84 (0.76 – 8.06)*	2.07 (0.63 – 6.69)	**0.0007**
Perimodiolar Array	1.48 (0.59 – 4.28)	2.02 (0.43 – 6.91)*	1.60 (0.69 – 3.12)	0.0002*

* *p* value (p < 0.05) of Kruskal Wallis test and Dunn’s test for post-hoc multiple comparisons (*)

Caption: Med = median; min = minimum; max = maximum; E16 = apical electrode; 16; E11 = medial electrode 11; E6 = basal electrode 6; tNRT= threshold Neural Response Telemetry; SOE = *Spread of excitation*; CL = current level; mm = millimeters; μV = microvolts

In straight arrays, Dunn’s post hoc analysis showed significant threshold differences between electrodes 6 and 16, and 11 and 16, with the lowest threshold at electrode 16. For both array types, significant SOE width differences were observed between electrodes 6 and 11, and 11 and 16, with the widest spread at electrode 11. Apical electrode 16 exhibited the narrowest SOE width in both groups (Table3).

## DISCUSSION

Since current spread arises from excitation of specific, potentially overlapping neural fiber populations, this study aimed to explore regional differences in current spread in the cochlea.

SOE measures differed significantly between straight and perimodiolar arrays, underscoring the need for separate analysis^(13,15-17)^.

The neural response threshold influences the choice of current required to obtain the SOE recording. In the present sample it was observed that tNRT thresholds were lower in apical regions for both array types, likely due to closer modiolus proximity of apical electrodes and eventual higher neural survival, which led to lower current levels used for the SOE function in that region^(11,23)^.

In addition, within straight arrays, significant threshold differences were observed between apical (electrode 16) and basal (electrode 6) electrodes. The literature points to several factors that explain the difference in eCAP thresholds in different regions of the cochlea, this may stem from electrode positioning relative to spiral ganglion cells or insertion trauma leading to fibrosis^(8,24)^.

The SOE curve peaks at the stimulating electrode; in tonotopically selective cochleae, amplitude is expected to diminish with increasing masker distance^(25)^. Greater SOE width may represent reduced selectivity, as remote fibers are recruited by the same stimulus.

SOE peak amplitudes were similar across cochlear regions and array types, indicating comparable ganglion cell recruitment despite regional threshold differences. It is also possible to interpret that the greater dispersion of excitation was not due to a greater number of fibers responding, but probably due to conductivity characteristics of the medium (perilymph) and the otic capsule^(6)^.

SOE width varied significantly between basal and medial, and medial and apical electrodes, with medial regions showing the broadest spread in both array types, a finding associated with poor speech recognition in CI users^(20)^.

Cochlear anatomy with a wider base and tapered apex^(3)^ may lead to asymmetric current spread, consistent with our results. Such differences can affect channel interaction, neural recruitment, loudness growth, and programming parameters.

Contrary to our findings, Einsen and Franck^(26)^ observed greater apical interaction in straight arrays, attributing this to higher neuronal density and excitability in the apex.

One aspect that may explain the different findings between studies concerns the variability of etiologies found in the present study sample, which may produce a different pattern of ganglion cell loss compared to a matched group with more uniform etiologies. Furthermore, it is not yet fully understood in the literature whether a higher density of excitable ganglion cells alone would influence channel interaction^(20)^.

In the present study, the width of the SOE was wider in the straight array when compared to the perimodiolar array in all the electrodes tested (6, 11 and 16). Coutinho da Silva et al.^(20)^ in a retrospective study of 323 ears implanted with different electrode arrays, analyzing separately the results of individuals with pre-lingual deafness and individuals with post-lingual deafness, also found a significant difference in SOE width between the straight and perimodiolar arrays, with wider spread in the straight array.

These data aligned with literature suggesting that proximity to the modiolus enhances neural excitability, reduces current needs for stimulation, and may minimize channel interaction^(27,28)^.

Understanding cochlear spread of excitation and its relationship with auditory physiology is crucial for refining CI programming and guiding future research. SOE influences neural recruitment and loudness perception, impacting current levels for comfort and the electrical dynamic range. Demonstrating regional differences in spread indicates that a single SOE measure is insufficient to define the dynamic field for the entire cochlea.

However, our study was limited by the lack of control or analysis of SOE across different etiologies, which could impact results. Etiological stratification is recommended for future studies to clarify inter-individual variability in electrical stimulation response.

## CONCLUSION

Although SOE amplitudes were similar across cochlear regions, implying comparable neural population recruitment, the medial region exhibited greater spread of excitation even in perimodiolar arrays. Objective tests like SOE are thus increasingly important for optimizing CI mapping and enabling more effective, individualized programming.
