# Supplementary material for: Is there a difference in the spread of excitation at different electrode locations along the cochlea?
Source: Codas. 2025 Oct 20;37(5):e20240090. doi: 10.1590/2317-1782/e20240090en (PMC12617284; doi:10.1590/2317-1782/e20240090en)
Supplement: Translation

# Translation: Existe diferença na dispersão de excitação ao longo da cóclea?

**DOI:** 10.1590/2317-1782/e20240090pt

**Authors:** Georgea Espindola Ribeiro, Ana Tereza de Matos Magalhães, Maria Valéria Schmidt Goffi Gomez, Carolina Gianzantti da Costa, Robinson Koji Tsuji, Rubens Vuono de Brito

**Affiliations:** 1 Departamento de Oftalmologia, Otorrinolaringologia e Cirurgia de Cabeça e Pescoço, Faculdade de Medicina, Universidade de São Paulo – USP - São Paulo (SP), Brasil.

**Keywords:** Audição, Surdez, Implante Coclear, Telemetria, Eletrodos, Resposta Neural, Dispersão da Corrente, Interação Entre Canais

## Abstract

**Objetivo:**

Identificar se existem diferenças na amplitude e largura da dispersão da excitação (spread of excitation - SOE) nas regiões apical, medial e basal da cóclea.

**Método:**

Estudo retrospectivo de corte transversal aprovado pela comissão de ética da instituição. Foram incluídos no estudo, adultos com surdez pós-lingual, submetidos ao implante coclear (IC), com presença de respostas neurais intraoperatórias, nos quais a SOE foi investigada em eletrodos representando as regiões basal (6), medial (11) e apical (16) da cóclea. Limiares da resposta neural, amplitudes de pico (µv) e largura da SOE em milímetros foram coletados e agrupados pelo modelo de feixe de eletrodos para análise pelos testes de Mann Whitney e Kruskal Wallis.

**Resultados:**

Foram selecionados 71 indivíduos, 27 implantados com feixe perimodiolar e 44 com feixe reto. Não houve diferenças significantes nos limiares da resposta neural e nas amplitudes do pico entre os eletrodos avaliados em ambos os grupos. Porém, a largura da SOE do eletrodo medial foi significantemente maior em ambos os feixes, seguida pela largura do eletrodo basal.

**Conclusão:**

Apesar da amplitude da SOE ser semelhante entre os diferentes locais ao longo do feixe de eletrodos, sugerindo recrutamento de populações neurais similares nas diferentes regiões da cóclea, foi encontrada maior dispersão de excitação na região medial mesmo nos eletrodos perimodiolares. Assim, o uso de testes objetivos será cada vez mais importante para auxiliar no mapeamento do IC, visando uma programação mais eficaz e individualizada.

## INTRODUÇÃO

A indicação do implante coclear (IC) é feita em casos de perda auditiva sensorioneural de grau severo e/ou profundo bilateral sem benefício com o uso de próteses auditivas convencionais^(1)^. O IC consiste na inserção de um feixe de eletrodos na escala timpânica sendo posicionado sob a membrana basilar e a lâmina espiral óssea^(2)^.

Os eletrodos inseridos na cóclea fornecem corrente elétrica para as fibras viáveis do nervo auditivo, distribuindo informações tonotópicas ao longo da cóclea sobre as informações espectrais do sinal acústico^(3)^. A percepção da intensidade (*loudness*), acontece pelo número de fibras ativadas (somação espacial) ou frequência dos impulsos nervosos (somação temporal)^(4)^. Dessa forma, o correto posicionamento dos eletrodos e proximidade modiolar são de suma importância para a estimulação elétrica eficaz^(5-7)^.

Pfingst et al.^(8)^ afirmaram que a estrutura anatômica da cóclea, bem como a distribuição das células ganglionares remanescentes é diferente ao longo de seu comprimento (porção basal, medial e ápice). Dessa forma, a estimulação dos eletrodos, ainda que projetados para respeitar a tonotopia, difere ao longo do feixe, sendo necessário maior ou menor nível de corrente a fim de se estimular as estruturas neurais em cada posição.

Sendo assim, o registro do potencial de ação composto do nervo auditivo (eCAP), obtido por meio da telemetria de resposta neural (NRT), permite investigar, tanto a partir da obtenção do limiar do eCAP quanto por meio de medidas avançadas, como a dispersão de excitação neural “*Spread of Excitation”* (SOE), o comportamento das fibras neurais nas diferentes posições dos eletrodos ao longo da cóclea^(6,9,10)^.

A dispersão de excitação neural pode ser medida a partir de método de mascaramento prévio, variando o estímulo mascarador ao longo dos eletrodos, mantendo fixo o estímulo no eletrodo prova e o eletrodo de registro. A captação da amplitude do potencial com a variação do estímulo mascarador em diferentes eletrodos gera uma curva denominada como SOE^(3,11)^. Pelo paradigma de subtração, a amplitude da resposta, após a subtração, será máxima quando o estímulo mascarador e prova recrutarem as mesmas fibras. É esperado que a amplitude da resposta após a subtração diminua com o distanciamento do eletrodo mascarador, pois as fibras que estão sendo estimuladas pelo eletrodo prova não estarão no período refratário, considerando que a estimulação do eletrodo mascarador não as atinge^(12)^. A curva formada tem sua máxima amplitude em torno do eletrodo prova, ou seja, onde o mascarador e o tom prova coincidem^(12)^ (Figura 1).

**Figura 1:** Esquema da SOE com técnica de mascaramento prévio adaptada de Abbas et al.^(12)^.

Legenda: (1) Painel direito, no traço A, o estímulo prova é apresentado e gera tanto o artefato quanto a resposta neural. Em B, o mascarador é apresentado com intervalo suficiente (MPI – intervalo mascarador-sonda) para que, quando o estímulo prova é apresentado, as fibras estejam em período refratário. O resultado são os artefatos do mascarador e prova e a resposta apenas do mascarador. Em C, o estímulo mascarador é apresentado e gera tanto um artefato quanto uma resposta neural. Em D, nenhuma estimulação é aplicada, apenas o amplificador é ligado. A subtração dos traçados resulta apenas na resposta neural ao estímulo prova

Idealmente, cada eletrodo intracoclear deve estimular apenas as fibras neurais mais próximas a ele, no entanto o campo elétrico gerado pelo eletrodo estimulador (prova) pode se espalhar no espaço intracoclear, e estimular também fibras mais distantes^(3,11)^. Quando a dispersão de corrente for muito ampla, pode haver influência na precisão com que cada canal apresente informações espectrais ao nervo auditivo^(13)^.

Na prática, quando ocorrer a dispersão de excitação, ampla, o usuário de IC pode apresentar dificuldade no reconhecimento de vogais e consoantes, podendo afetar também a percepção do *pitch*, impactar na sensação de *loudness* e nos níveis de corrente na programação, levando a resultados limitados no processo da reabilitação auditiva^(14)^.

Dessa forma, entender como ocorre o processo de dispersão de corrente em diferentes pontos do feixe de eletrodo poderá auxiliar na identificação de regiões da cóclea que têm ausência ou poucos neurônios estimuláveis, o que posteriormente favorecerá na otimização de diferentes parâmetros para cada indivíduo ao longo das programações.

Outro aspecto que pode influenciar na medida da SOE é o tipo do feixe de eletrodos. Embora Kopsch et al.^(15)^ tenham reportado não encontrar diferença da SOE entre feixes retos e perimodiolares, o estudo contou somente com dois indivíduos usuários de feixe reto e 57 perimodiolares. Já Kim et al.^(16)^ observaram que os eletrodos de feixe reto apresentavam maior largura da SOE em relação ao de feixe perimodiolar. Os autores indicaram menor sobreposição na excitação neural e interação de canal no feixe de eletrodos periomodiolar. Berg et al.^(17)^ ao realizarem a medida da SOE no momento intraoperatório em uma população implantada com eletrodos de feixe reto e perimodiolar observaram que a largura da SOE, aumentava à medida que se aumentava a distância dos contatos dos eletrodos ao modíolo, tal efeito foi encontrado apenas para os feixes de eletrodos perimodiolares. Para o feixe de eletrodos retos, os autores observaram maior dispersão de corrente com o aumento da profundidade de inserção do feixe.

Com relação aos valores da SOE em pontos distintos da cóclea, Xi et al.^(13)^ e Rader et al.^(18)^ observaram que a região apical apresentou maior dispersão de corrente do que a região basal, e justificam tal achado devido a geometria assimétrica da cóclea, apresentando no ápice, uma estrutura espiral mais afilada e com maior densidade neuronal. Porém, Söderqvist et al.^(14)^ encontraram maior dispersão de corrente da SOE na região medial da cóclea. Os autores argumentam que esses resultados podem ser atribuídos ao tamanho relativo da escala timpânica e a uma possível má condutividade do tecido ao redor da rampa timpânica, levando a perda de corrente pela janela redonda, causando o rápido decaimento do campo elétrico.

Os resultados apresentados apontam que diferentes feixes de eletrodos podem levar a diferentes perfis da SOE. Dessa forma, o presente estudo hipotetiza que tanto a distância ao modíolo quanto a sobrevivência neural podem influenciar a dispersão de corrente elétrica, levando a crer que essa dispersão seja menor nos eletrodos perimodiolares em relação aos eletrodos de feixe reto, devido à sua maior proximidade ao modíolo. Da mesma forma, espera-se que a dispersão de corrente seja menor na região apical para o feixe perimodiolar pelo mesmo motivo, enquanto na região basal da cóclea a dispersão de corrente elétrica deve ser semelhante entre os feixes, por estarem afastados do modíolo.

Sendo assim, o objetivo do presente estudo foi identificar se existem diferenças na amplitude e largura de dispersão da SOE entre as regiões apical, medial e basal da cóclea, tanto para eletrodos com feixe reto quanto perimodiolar.

## MÉTODO

Trata-se de um estudo retrospectivo de corte transversal aprovado pelo Comitê de Ética da instituição (número CAAE: 03409212.8.0000.0068) e todos os participantes assinaram o TCLE (termo de consentimento livre e esclarecido).

Os dados foram obtidos por meio da análise de prontuários e de registros intraoperatórios de indivíduos implantados pelo Grupo de Implante Coclear do Hospital das Clínicas da Faculdade de Medicina da Universidade de São Paulo.

### Casuística

Foram selecionados registros intraoperatórios do banco de dados do grupo de implante coclear, referentes ao período de janeiro de 2016 e junho de 2021, com base nos seguintes critérios de inclusão: indivíduos implantados com dispositivos *Nucleus® (Cochlear Corporation*®) com eletrodos de feixes perimodiolares (CI 24RE CA, CI532, CI632) e retos (CI422, CI522 e CI622); adultos (≥ 18 anos de idade) com surdez de instalação pós-lingual, que apresentavam registro da pesquisa do limiar da resposta neural (tNRT) e SOE nos eletrodos 6, 11 e 16, pela técnica do mascaramento prévio^(12,19)^ no momento intraoperatório.

Os critérios de exclusão foram: indivíduos com etiologia de neuropatia ou alterações anatômicas na cóclea, presença de estimulação do nervo facial durante a NRT, ausência de NRT, inserção parcial dos eletrodos ou presença de dobra da ponta do feixe de eletrodos (*tip foldover).*

As seguintes informações foram obtidas a partir do prontuário dos participantes: idade na cirurgia, etiologia, instalação da surdez e tipo de feixe de eletrodos. No *software* de registro da resposta neural foi analisado novamente e coletado o valor do tNRT, a medida da amplitude do pico da resposta neural em microvolts (µv), medida da largura de dispersão em milímetros (mm), no ponto 0,75 da curva e nível de corrente utilizado para o registro.

### Procedimentos

O protocolo para a gravação do eCAP foi realizado através do *software CustomSound EP 3.0*®, instalado em um microcomputador portátil acoplado à interface de programação portátil (PPS, do inglês *portable programming system*) e ao processador de fala modelo *Nucleus*® 5 ou superior, ambos produzidos pela *Cochlear Corporation®.* Logo após a inserção dos eletrodos na cóclea e com o paciente anestesiado, foi efetuada a avaliação da integridade do IC (telemetria de impedâncias), seguido da NRT (tNRT e SOE).

Para o registro do eCAP, um trem de pulsos elétricos foi enviado nos eletrodos intracocleares 6, 11 e 16 correspondentes a diferentes regiões cocleares, respectivamente porção basal, medial e apical, e a resposta neural foi registrada por um eletrodo adjacente. Convencionalmente foi formado uma onda com pico inicial negativo seguido por um pico positivo, denominadas de N1 e P1, respectivamente. A amplitude do eCAP foi determinada pela diferença de voltagem entre as ondas N1 e P1, sendo que o próprio programa aplicou um ajuste de regressão linear sobre a curva de crescimento de amplitude, determinando o tNRT e a inclinação dessa curva (função I/O) para cada eletrodo estudado. Dessa forma, o limiar foi identificado como a menor corrente de estímulo em que se obteve a resposta neural^(12)^.

O protocolo para coleta da SOE foi realizado com 10 *current level* (CL) ou mais, acima da tNRT^(20)^. Para as gravações da SOE foi aplicada uma taxa de estimulação de 40 Hz e intervalo entre o mascarador e os estímulos da sonda de 400 μs, e os valores foram obtidos variando o eletrodo mascarador. Sendo extraída informações referentes a amplitude do pico da curva (µv), medida da largura de dispersão em milímetros (mm), no ponto 0,75 da curva e nível de corrente utilizado para o registro da SOE (Figura 2).

**Figura 2:** Tela do *software Custom Sound* EP mostrando o registro da dispersão de excitação neural (Spread of Excitation = SOE), com a medida da largura da dispersão calculada em milímetros (mm) no ponto 75% da curva. A chave na horizontal foi traçada para ilustrar a largura medida, no exemplo, 2,5 mm

A seleção da amostra estudada foi de conveniência, com todos os participantes implantados que atenderam os critérios de inclusão no período do estudo.

Para análise dos resultados, os participantes foram agrupados e comparados nos feixes retos e perimodiolares. Devido à distribuição não normal dos dados, à aleatoriedade e à independência da amostra, isto é, pelo estudo tratar-se de uma amostra por conveniência, na qual os participantes foram agrupados e comparados entre os feixes retos e perimodiolares, sem correspondência direta entre os indivíduos de cada grupo, a análise estatística foi realizada por meio do teste não paramétrico de Mann-Whitney para a comparação entre os tipos de feixes. Para a análise entre as diferentes regiões do feixe de eletrodos (6, 11 e 16), utilizou-se o teste de Kruskal-Wallis, com o teste de Dunn para comparações múltiplas post hoc^(21,22)^ por meio do *software* BioEstat 5.0, adotando-se nível de significância de 5% em todos os testes.

## RESULTADOS

Dos 177 registros intraoperatórios obtidos, 71 atenderam aos critérios de inclusão do estudo. Na Tabela 1 encontra-se a distribuição demográfica dos dados obtidos.

**Tabela 1:** Distribuição demográfica da amostra estudada

	Eletrodo reto (n=44)	Eletrodo perimodiolar (n=27)
Média de idade (anos)	52 (mín 19/máx 81)	42 (mín 19/máx 74)
Tipo de feixe (N)		
Eletrodo reto		
CI 422	40	-
CI 522	3	-
CI 622	1	-
Eletrodo Perimodiolar		
CI 532	-	14
CI 24RECA	-	12
CI 632	-	1
Etiologia (N)		
Desconhecida	27	14
Otosclerose	5	4
Meningite	4	-
Traumatismo cranioencefálico	3	2
Ototoxicidade	2	1
Infecção		2
Mondini	-	2
Rubéola	1	1
Autoimune	1	-
Perda auditiva súbita	-	1
Otite média crônica	1	-

Legenda: N = número de orelhas; min = mínimo; max = máximo

Os limiares da tNRT foram estatisticamente diferentes entre os feixes reto e perimodiolar somente no eletrodo 6 (basal). As amplitudes foram semelhantes em todas as regiões da cóclea entre os feixes reto e perimodiolar. A largura da SOE foi estatisticamente diferente entre os feixes reto e perimodiolar, mostrando maior dispersão de excitação nos feixes retos para todas as regiões da cóclea (Tabela 2).

**Tabela 2:** Mediana das variáveis estudadas e a comparação entre os feixes reto e perimodiolar pelo teste de Mann Whitney

	Feixe reto (N = 44)	Feixe perimodiolar (N = 27)	*p* *
tNRT (CL)	Md. (min/máx)	Md. (min/máx)	
E16	191 (142 – 232)	184 (162 – 209)	0,3816
E11	200 (177 – 234)	202 (165 – 223)	0,6608
E6	206 (183 – 237)	198 (155 – 218)	0,0050*
SOE amplitude (μV)			
E16	83,33 (11,95 – 265,71)	66,94 (12,98 – 233,95)	0,5242
E11	68,31 (10,24 – 234,63)	67,62 (10,59 – 200,14)	0,6109
E6	67,62 (18,10 – 283,81)	57,03 (20,49 – 209,36)	0,3769
SOE largura (mm)			
E16	2,31 (0,88 – 5,31)	1,58 (0,59 – 4,38)	0,0007*
E11	3,30 (1,01 – 8,06)	2,44 (0,59 – 3,73)	0,0003*
E6	2,02 (0,63 – 6,69)	1,68 (0,69 – 3,12)	0,0429*

* *P* value pelo teste de Mann Whitney

Legenda: N = número feixes de eletrodos; Md. = mediana; min = mínimo; máx = máximo; E16 = eletrodo apical 16; E11 = eletrodo medial 11; E6 = eletrodo basal 6; tNRT = limiar da resposta neural; SOE = *Spread of excitation*; CL = unidade em nível de corrente; mm = milímetros; μV = microvolts

Ao avaliar os limiares tNRT, observaram-se diferenças entre os eletrodos 6, 11 e 16 tanto para o feixe periomodiolar quanto para o feixe reto, havendo menor limiar na região apical (eletrodo 16), para ambos os feixes de eletrodos (Tabela 3).

**Tabela 3:** Resultados dos parâmetros estudados nos grupos implantados com feixe reto e perimodiolar, analisados pelo teste de Kruskal Wallis

	E16	E11	E6	*p* *
tNRT (CL)	Md. (min/máx)	Md. (min/máx)	Md. (min/máx)	
Feixe reto	189 (142 – 232)*	200 (156 – 235)	205 (183 – 237)	**<0,0001**
Feixe perimodiolar	186 (155 – 209)	194 (159 – 229)	197 (155 – 221)	0,1281
SOE amplitude do pico (μV)				
Feixe reto	95 (21 – 265)	69 (19 – 271)	67 (24 – 300)	0,6181
Feixe perimodiolar	65 (12 – 256)	68 (14 – 247)	56 (21 – 209)	0,6481
SOE largura (mm)				
Feixe reto	2,34 (0,88 – 5,31)	2,84 (0,76 – 8,06)*	2,07 (0,63 – 6,69)	**0,0007**
Feixe perimodiolar	1,48 (0,59 – 4,28)	2,02 (0,43 – 6,91)*	1,60 (0,69 – 3,12)	0,0002*

* *p* value (p < 0,05) pelo teste de Kruskal Wallis e teste de Dunn para comparações múltiplas post-hoc (*)

Legenda: Md. = mediana; min = mínimo; máx = máximo; E16 = eletrodo apical 16; E11 = eletrodo medial 11; E6 = eletrodo basal 6; tNRT = limiar da resposta neural; SOE = *Spread of excitation*; CL = unidade em nível de corrente; mm = milímetros; μV = microvolts

Não houve diferenças significantes entre as amplitudes do pico da SOE (µv), entre os eletrodos pesquisados para ambos os feixes de eletrodos (Tabela 3). No feixe reto, a análise post hoc pelo teste de comparações múltiplas de Dunn mostrou diferença significante no limiar da resposta neural entre os eletrodos 6 e 16, e entre os eletrodos 11 e 16, sendo o menor limiar observado no eletrodo 16. Tanto para o feixe reto como para o feixe perimodiolar, a análise post hoc, realizada por meio do teste de comparações múltiplas de Dunn, revelou diferença significante na largura da dispersão de excitação entre os eletrodos 6 e 11, e entre os eletrodos 11 e 16, sendo a maior largura observada no eletrodo 11. Nota-se também a semelhança de uma menor largura da SOE (mm) nos eletrodos apicais (eletrodo 16) em ambos os grupos (Tabela 3).

## DISCUSSÃO

Sabendo-se que a dispersão de corrente entre os eletrodos ocorre porque cada eletrodo excita uma população de fibras neurais específica e que essas populações neurais podem se sobrepor, o objetivo do presente estudo foi verificar se existem diferenças de dispersão de corrente em distintas regiões da cóclea.

Para estudar a dispersão de excitação por meio da SOE, observou-se que as medidas são significativamente diferentes entre os eletrodos de feixe reto e perimodiolar, o que evidencia a necessidade de analisá-los separadamente^(13,15-17)^.

Para o registro da SOE, é feita a pesquisa do limiar do eCAP por meio da NRT, e na amostra estudada observou-se que, para os diferentes tipos de feixe (reto e perimodiolar) o limiar do eCAP foi menor na região apical da cóclea. Uma justificativa para tal achado é atribuída pelos eletrodos dessa região estarem mais próximos ao modíolo do que outros eletrodos, excitando dessa forma um maior número de fibras, além do mais, existe a presunção de que há uma maior sobrevivência neural nessa região da cóclea havendo consequentemente um menor limiar eletrofisiológico^(11,23)^.

Além disso, no feixe reto observou-se que a tNRT é estatisticamente diferente entre o eletrodo 16 (apical) e 6 (basal). A literatura aponta alguns fatores que justificam a diferença de limiar do eCAP em distintas regiões da cóclea, como por exemplo, a maneira pela qual o feixe de eletrodos situa-se dentro da cóclea em relação às células ganglionares espirais e eventual trauma gerado no momento da inserção podendo influenciar na formação de fibrose^(8,24)^.

Sabe-se que a função da SOE evidencia a sobreposição de populações neurais recrutadas pelo eletrodo mascarador em diferentes posições junto ao eletrodo estimulador. Sendo assim, a curva resultante da SOE tem sua amplitude máxima na resposta neural referente ao eletrodo estimulador. Em cócleas tonotopicamente seletivas, é esperado que a amplitude da SOE diminua com o aumento da distância entre o mascarador e o estimulador^(25)^. Ou seja, quanto maior a largura da dispersão de excitação, menos seletiva é aquela região, pois significa que fibras remotas ao eletrodo estimulador estão respondendo ao mesmo estímulo.

Ao se analisar a amplitude do pico da SOE, observou-se resultados semelhantes entre os locais da cóclea para ambos os feixes. Esse resultado indica que, embora os limiares da NRT apresentem diferenças entre as regiões basal, medial e apical, o recrutamento de células ganglionares, representado pela amplitude, foi semelhante. Também é possível interpretar que a maior dispersão de excitação não foi por maior número de fibras respondendo, mas provavelmente por características de condutibilidade do meio (perilinfa) e da cápsula ótica^(6)^.

Na amostra estudada, nos feixes retos e perimodiolares, a largura da SOE foi estatisticamente diferente entre os eletrodos basais e mediais (6 e 11), e entre os eletrodos mediais e apicais (11 e 16), mostrando que a largura da SOE no eletrodo medial é maior e difere das demais. Esse fato demonstrou influenciar o reconhecimento de fala de usuários adultos de IC^(20)^.

A anatomia coclear apresenta uma geometria mais larga em sua base e uma morfologia afilada no ápice^(3)^, podendo-se levantar a hipótese de uma dispersão de corrente de forma assimétrica das fibras nervosas, justificando os dados obtidos nesse estudo. Essas diferenças podem impactar não só a interação entre os canais, mas também no recrutamento neural que, por sua vez pode influenciar a sensação de crescimento de *loudness* e os níveis de estimulação da programação do IC.

Diferentemente dos achados desta pesquisa, Einsen e Franck^(26)^ encontraram maior interação nos eletrodos da extremidade apical em comparação com a extremidade basal nos feixes retos. Os autores justificaram tal feito devido a porção apical apresentar maior densidade e excitabilidade das células ganglionares sobreviventes, o que contribuiria para uma maior dispersão de corrente nessa região. Um aspecto que pode justificar os diferentes achados entre as pesquisas, diz respeito a variabilidade de etiologias encontradas na amostra, o que pode produzir um padrão diferente de perda de células ganglionares.

Ainda nesse estudo, observou-se que a largura da SOE foi maior no feixe reto quando comparado ao perimodiolar em todos os eletrodos testados (6, 11 e 16). Coutinho da Silva et al.^(20)^ em estudo retrospectivo com 323 orelhas implantadas com diferentes feixes de eletrodos, analisando separadamente os resultados de indivíduos com surdez pré e indivíduos com surdez pós-lingual, também encontraram diferença significante da largura da SOE entre o feixe reto e perimodiolar, ou seja, a largura da SOE foi maior no feixe reto.

Essas informações vão de encontro com dados da literatura que apontam que, quanto mais próximo o feixe de eletrodos estiver junto ao modíolo, melhor será a excitabilidade das fibras neurais, portanto, menor será a corrente elétrica necessária para a estimulação neural, e supõe-se uma menor interação entre os eletrodos^(27,28)^.

Desta forma, a compreensão sobre a dispersão da corrente ao longo da cóclea e sua correlação com a fisiologia auditiva é de suma importância para auxiliar nos parâmetros das programações do IC e direcionar futuros estudos, pois a SOE pode influenciar no recrutamento neural e consequentemente na sensação de intensidade (*loudness*). Isso impacta diretamente nos valores das medidas de corrente necessária para provocar o *loudness* correspondente ao nível de conforto e o campo dinâmico elétrico (diferença entre nível máximo e mínimo de estimulação elétrica). A demonstração de que a dispersão é diferente ao longo das regiões da cóclea mostra que uma única medida de dispersão não seria suficiente para identificar o campo dinâmico de toda a cóclea.

No entanto, vale ressaltar que, neste estudo, houve limitações em relação à ausência de controle e análise da SOE para diferentes etiologias, o que poderia impactar diretamente os resultados. A falta de agrupamento ou estratificação das etiologias impediu a identificação de possíveis variáveis que poderiam influenciar a resposta da SOE, como variações anatômicas ou fisiológicas associadas a causas específicas de perda auditiva. Dessa forma, sugere-se a necessidade de estudos futuros que estratifiquem as etiologias para uma melhor compreensão das diferenças interindividuais na resposta à estimulação elétrica.

## CONCLUSÃO

Apesar da amplitude da SOE ser semelhante entre os diferentes locais ao longo do feixe de eletrodos, sugerindo recrutamento de populações neurais similares nas diferentes regiões da cóclea, foi encontrada maior dispersão de excitação na região medial mesmo nos eletrodos perimodiolares. Assim, o uso de testes objetivos será cada vez mais importante para auxiliar no mapeamento do IC, visando uma programação mais eficaz e individualizada.
